# Impact of In-House 3D-Printed Models on Re-Operation Rates and Volumetric Precision in Orbital Floor Reconstruction: A Comparative Study

**DOI:** 10.3390/jcm15082822

**Published:** 2026-04-08

**Authors:** Ilze Prikule, Ieva Bagante, Oskars Radzins, Girts Salms

**Affiliations:** 1 Department of Oral and Maxillofacial Surgery, Riga Stradins University, LV-1007 Riga, Latvia; 2 Centre of Oral and Maxillofacial Surgery, Pauls Stradins Clinical University Hospital, LV-1002 Riga, Latvia; 3 Baltic Biomaterial Centre of Excellence, Headquarters at Riga Technical University, LV-1048 Riga, Latvia; 4 Institute of Stomatology, Riga Stradins University, LV-1007 Riga, Latvia

**Keywords:** orbital fractures, three-dimensional printing, reconstructive surgical procedures, reoperation, orbital volume, orbital reconstruction

## Abstract

**Background/Objectives**: Reconstruction of orbital floor fractures remains surgically challenging due to limited intraoperative visibility and complex anatomy. Inaccurate implant placement often leads to persistent complications and the need for a revision surgery. This study evaluated the clinical accuracy and re-operation rates of a preoperative 3D-printed model-assisted technique compared to the conventional intraoperative free-hand mesh bending method. **Methods**: A comparative ambispective study was conducted on 74 patients with isolated orbital floor fractures. The control group (*n* = 34, retrospective) underwent reconstruction using intraoperatively formed titanium meshes. In the study group (*n* = 40, prospective), patient-specific 3D-printed models, created by mirroring the healthy contralateral orbit, were used for preoperative mesh adaptation. Primary outcomes included the rate of revision surgery due to implant malposition, changes in orbital volume, and postoperative diplopia. **Results**: The 3D model group demonstrated a significantly lower rate of revision surgery compared to the control group. In the retrospective group, 5 patients (15%) required reoperation due to implant malposition, whereas no patients (0%) in the prospective 3D group required secondary intervention (*p* = 0.017). While both techniques effectively restored orbital volume, the 3D group showed greater volumetric precision with less variance. The mean volume difference in the affected orbit was 3078 ± 2204 mm^3^ in the control group, compared to 2390 ± 1893 mm^3^ in the study 3D group. At the 6-month follow-up, persistent diplopia was observed in 12% of the control group compared to only 3% in the study group. **Conclusions**: The use of in-house 3D-printed models for preoperative mesh forming significantly enhances surgical precision and eliminates the need for revision surgery due to implant malposition. This workflow offers a cost-effective, predictable, and accessible alternative to expensive patient-specific implants (PSIs) or intraoperative navigation systems, improving patient safety and long-term clinical outcomes.

## 1. Introduction

The management of orbital fractures represents a significant and current topic within the field of maxillofacial surgery, with a growing body of literature reflecting the ongoing search for the optimal reconstruction technique. Isolated orbital floor fractures are among the most common midfacial injuries, representing approximately 4–16% of all facial fractures [1]. The primary goal of surgical management is to restore the anatomical orbital volume and correct the shape of the orbital cavity to treat enophthalmos, diplopia, and ocular motility restriction. However, the surgical methods to achieve this vary widely. To improve precision, surgeons today employ a wide range of technologies, including virtual surgical planning (VSP), patient-specific implants (PSI), intraoperative navigation, and intraoperative CT imaging, either individually or in combination. Despite these technological advancements, the reconstruction of the orbital floor remains surgically challenging, due to the complex anatomy of the orbital floor and the limited surgical exposure provided by standard minimally invasive approaches—transconjunctival or subciliary—which are favoured for their ability to minimize visible scarring but restrict the visual field.

Conventionally, reconstruction is performed using standard titanium meshes that are adapted intraoperatively. While effective, this method relies heavily on the surgeon’s subjective visual assessment and experience. Inadequate adaptation or incorrect placement of the implant can lead to significant complications. Recent studies indicate that the use of a standard titanium mesh is associated with a reoperation rate of up to 31.8% in long-term follow-up, mostly due to implant malposition [2]. To improve accuracy, advanced technologies such as intraoperative navigation, intraoperative computed tomography (CT), and patient-specific implants (PSIs) have been introduced [3,4,5]. However, despite their widespread availability, factors such as high cost, long production time, and logistical barriers limit their routine use in many centres. A recent survey of maxillofacial units in Switzerland, Germany, and Austria revealed that while 48% of clinics have access to 3D printers, the majority still rely on conventional methods due to these barriers [6].

A promising, cost-effective alternative is the in-house fabrication of 3D-printed anatomical models. By mirroring the healthy contralateral orbit, a sterilisable template can be created to prebend the standard titanium meshes before surgery. This approach aims to combine the precision of PSIs with the accessibility and low cost of standard implants, as well as the safety and precision of digital planning, making it a viable option for a wider range of hospitals. Three-dimensional technologies are also widely used in other medical fields, such as traumatology and orthopaedic. The structured 3D-assisted workflow showed improved anatomical understanding and surgical precision, remarkable accuracy, and cost-effectiveness that support the advancement and efficiency of clinical practice [7,8].

This study aimed to compare the clinical and radiological outcomes of orbital floor reconstruction using conventional intraoperative mesh bending versus preoperative mesh bending with a 3D-printed model technique. Specifically, the study assessed the precision of orbital volume restoration, the incidence of postoperative diplopia, and the rate of revision surgery required for implant correction. The hypothesis was that implementing preoperative bending of the standard titanium mesh for orbital reconstruction on the corresponding 3D-printed patient model would lead to improved orbital volume symmetry after surgery and reduced occurrence of double vision while simultaneously restoring facial aesthetics.

## 2. Materials and Methods

### 2.1. Study Design

This study performed at the Centre of Oral and Maxillofacial Surgery, Pauls Stradins Clinical University Hospital (P. Stradins CUH) in Riga, Latvia, was designed as a single-centre, non-randomized ambispective comparative cohort study. This study design was selected as it integrates both retrospective and prospective components within a single analytical framework. The control group, comprising patients treated with conventional intraoperative free-hand mesh bending, was evaluated retrospectively using historical clinical and radiological records. The study group, consisting of patients treated using the in-house 3D-printed models, was enrolled and assessed prospectively following the implementation of this technological protocol at our institution. The outcomes of the prospectively treated cohort were compared with those of the historical control group. The study protocol adhered to the Declaration of Helsinki and was approved by the Ethics Committee of Riga Stradins University (No. 2-PĒK-4/132/2022, approved 9 March 2022). The Ethics committee approval served as the foundational approval for the study design, authorising both the retrospective data analysis (2018–2021) and the initiation of the prospective study phase, which started on 9 March 2022, with its patient collection in the hospital. The study is registered in the ISRCTN registry (reference number ISRCTN12793225). Written informed consent was obtained from all patients in the prospective group. Data were anonymized, coded, and stored electronically in accordance with local data protection regulations.

### 2.2. Patient Selection

The study population consisted of two groups of adult patients treated for isolated orbital floor fractures. The retrospective group (control) included all consecutive patients treated between 1 January 2018 and 31 December 2021. The prospective group (study) included consecutive patients operated between 9 March 2022 and 30 September 2023.

The inclusion criteria were as follows: (1) adult patients (>18 years); (2) diagnosis of an isolated orbital floor fracture; (3) surgical reconstruction performed using a titanium mesh (either standard free-hand or 3D model-assisted); and (4) complete medical records, including pre- and postoperative ophthalmologic examinations and high-quality computed tomography (CT) scans (slice thickness ≤ 0.625 mm). The exclusion criteria were: (1) combined facial bone fractures (e.g., zygomaticomaxillary complex fractures); (2) orbital floor reconstruction using materials other than a single titanium mesh (e.g., absorbable membranes); (3) missing or insufficient quality of pre- or postoperative CT data; and (4) acute ophthalmological emergencies requiring immediate surgical intervention.

### 2.3. Surgical Protocol

All patients underwent surgical reconstruction under general anaesthesia. Standard surgical access to the orbital floor was achieved primarily through a transconjunctival or subciliar approach according to the surgeons and patients’ preferences.

**Figure 1:**
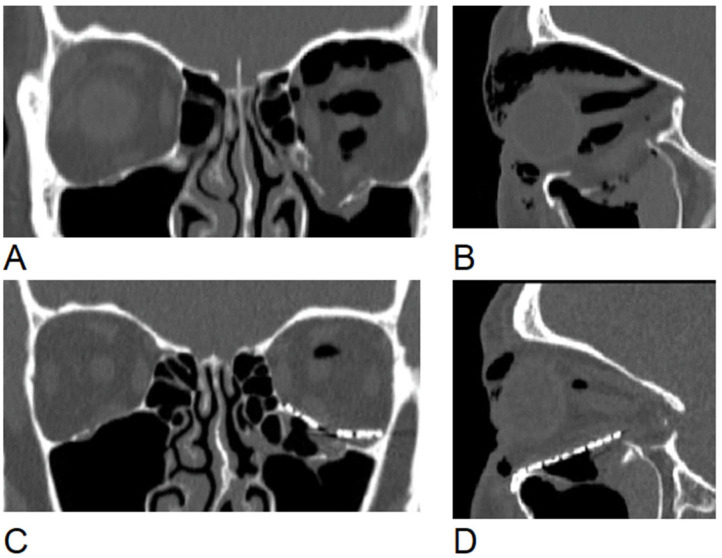
Computed tomography images of a patient from the retrospective group. (**A**,**B**)—preoperative view, (**C**,**D**) postoperative view.

In the retrospective group, reconstruction was performed using a standard intraoperative free-hand bending technique. The titanium mesh (KLS Martin, Jacksonville, FL, USA) was manually bent and adapted intraoperatively to fit the orbital defect. The mesh was inserted and adjusted based purely on the surgeon’s direct visual assessment and tactile feedback of the anatomical landmarks (Figure 1). To verify the adequacy of the reconstruction and ensure that no extraocular muscles or peri-orbital tissues were entrapped beneath the implant, a forced duction test was routinely performed prior to closure. No intraoperative navigation or 3D imaging was used; instead, radiological verification was achieved via a routine CT scan performed on the first postoperative day.

**Figure 2:**
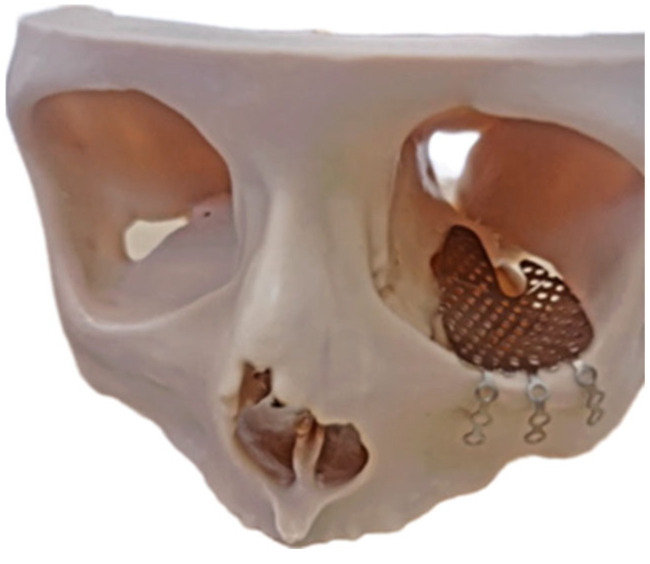
Three-dimensional-printed model for left orbital floor reconstruction with pre-bent KLS Martin titanium mesh.

**Figure 3:**
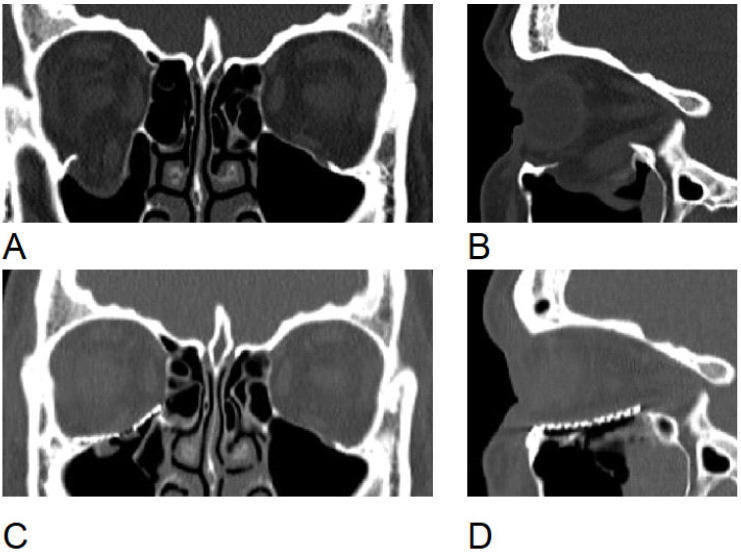
Computed tomography images of a patient from the prospective group. (**A**,**B**)—preoperative view, (**C**,**D**) postoperative view.

In the prospective group, step-by-step digital workflow was performed. (1) Image acquisition and virtual modelling: Preoperative high-resolution CT data (in DICOM format) were imported into Mimics software (Version 26.0, Materialise, Leuven, Belgium) to generate a virtual 3D model of the affected facial skeleton. (2) Segmentation and mirroring: The ideal reconstruction shape was established by mirroring the healthy contralateral orbit across the midsagittal plane. Since head positioning in preoperative scans often deviates from the ideal, the symmetry plane was adjusted manually. The final virtual models of the reconstructed orbit were then exported as Standard Tessellation Language (STL) files in both native and mirrored models. (3) Rapid prototyping and post-processing: STL files were transferred to an Asiga PRO 4K80 printer (Asiga, Sydney, Australia) and manufactured using a polymer light-cure resin (Asiga DentaMODEL, Sydney, Australia). Post-processing involved washing the models in isopropanol to clean excess unpolymerized resin, followed by the precise removal of support structures using abrasive cutters. The final polymerisation was achieved by curing the models in an Otoflash pulsing unit (600 flashes, or 10 flashes/second for 60 s). (4) Preoperative mesh contouring: A standard titanium mesh (KLS Martin, Jacksonville, FL, USA) was manually pre-bent and adapted onto the 3D-printed physical model to precisely match the mirrored anatomical landmarks of the orbital floor (Figure 2). (5) Sterilization: Prior to surgery, the customized titanium mesh underwent standard metal instrument sterilization, while both 3D-printed skull models were sterilized in a gas autoclave at 55 °C. During surgery, before final placement, the pre-bent mesh was re-checked intraoperatively against the sterile 3D-printed skulls to ensure no deformation had occurred during handling. The mesh was then inserted into the orbital floor and rigidly fixed with 5 mm titanium screws. Postoperatively, the physical 3D prototypes were anonymized and stored at the Baltic Biomaterials Centre of Excellence in accordance with the Latvian Cabinet of Ministers Regulation No. 265 regarding the storage of medical documentation. After the surgery, patients had a control CT of the orbits according to the already existing standard protocol (Figure 3).

### 2.4. Clinical and Radiological Assessment

The clinical evaluation was performed at 1–2 weeks, 2 months, and 6 months (if necessary) postoperatively. The key outcome parameters included enophthalmos, diplopia, and ocular motility. The presence of diplopia and eye motility was determined with subjective separation of double images in the nine positions of gaze with simple method (the ‘follow my-finger’ test) [5]. Visual fields were also recorded. The exophthalmos and enophthalmos were evaluated in CT and clinically. A difference of more than 2 mm was considered significant.

**Figure 4:**
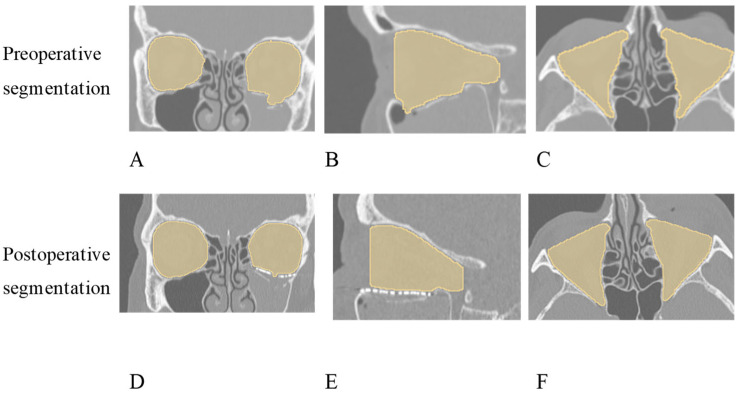
Computed tomography images of a patient treated with a pre-bent titanium mesh. (**A**)—Pre-operative coronal series, (**B**)—Pre-operative sagittal series, (**C**)—Preoperative axial series (**D**)—Postoperative coronal series, (**E**)—Postoperative sagittal series, and (**F**)—Postoperative axial series.

Orbital volume analysis was performed using 3D Slicer software (version 5.0.3, open source) on pre- and post-operative CT datasets exported in DICOM format. Semi-automatic segmentation was utilized to quantify and compare the orbital volumes (mm^3^) between the affected and healthy contralateral sides, both preoperatively and postoperatively. To accurately isolate the orbital bony cavity from surrounding structures, a combination of global standard bone-density Hounsfield Unit (HU) thresholding and manual slice-by-slice editing was applied. To ensure inter-patient reproducibility, the anatomical boundaries of the virtual orbital space were standardized for all measurements: (1) anterior boundary: defined by a strait virtual plane in the axial view connecting the anterior lacrimal crest medially to the lateral orbital rim, deliberately excluding the lacrimal groove and canal from the total volume, (2) posterior boundary: set at the orbital apex, specifically defined as the most distal bony edge of the optic canal just before its transition into the cranial fossa, (3) superior, medial, and lateral boundaries: delineated by their intact bony walls, and (4) inferior boundary: in the presence of a fracture, the preoperative volume was measured by tracing the herniated orbital contents downwards into the maxillary sinus. To evaluate the surgically reconstructed volume (and to establish the theoretical “ideal” baseline), the inferior boundary was defined by digitally as a line between the remaining bony structures of the defect across the coronal and sagittal planes. Postoperatively, the reconstructed inferior volumetric boundary was strictly defined by the radio-opaque contour of the implanted titanium mesh (Figure 4A–F).

### 2.5. Statistical Analysis

Statistical analysis was performed using Jamovi software (Version 2.7). Descriptive statistics were used to summarize the data. Intra-rater reliability was assessed by calculating the intraclass correlation coefficient (ICC) from five randomly selected cases (10 orbits: 5 fractured, 5 unaffected) for which the volume quantification process was repeated after one month. Differences in orbital volumes between the healthy and affected sides (pre- and postoperative surgery) were analysed using the Wilcoxon signed-rank test. Categorical variables, specifically the rate of postoperative complications and the need for surgical re-operation, were compared between the control and study groups using Fisher’s exact test. A *p*-value of <0.05 was considered as statistically significant.

## 3. Results

### 3.1. Patient Characteristics

**Figure 5:**
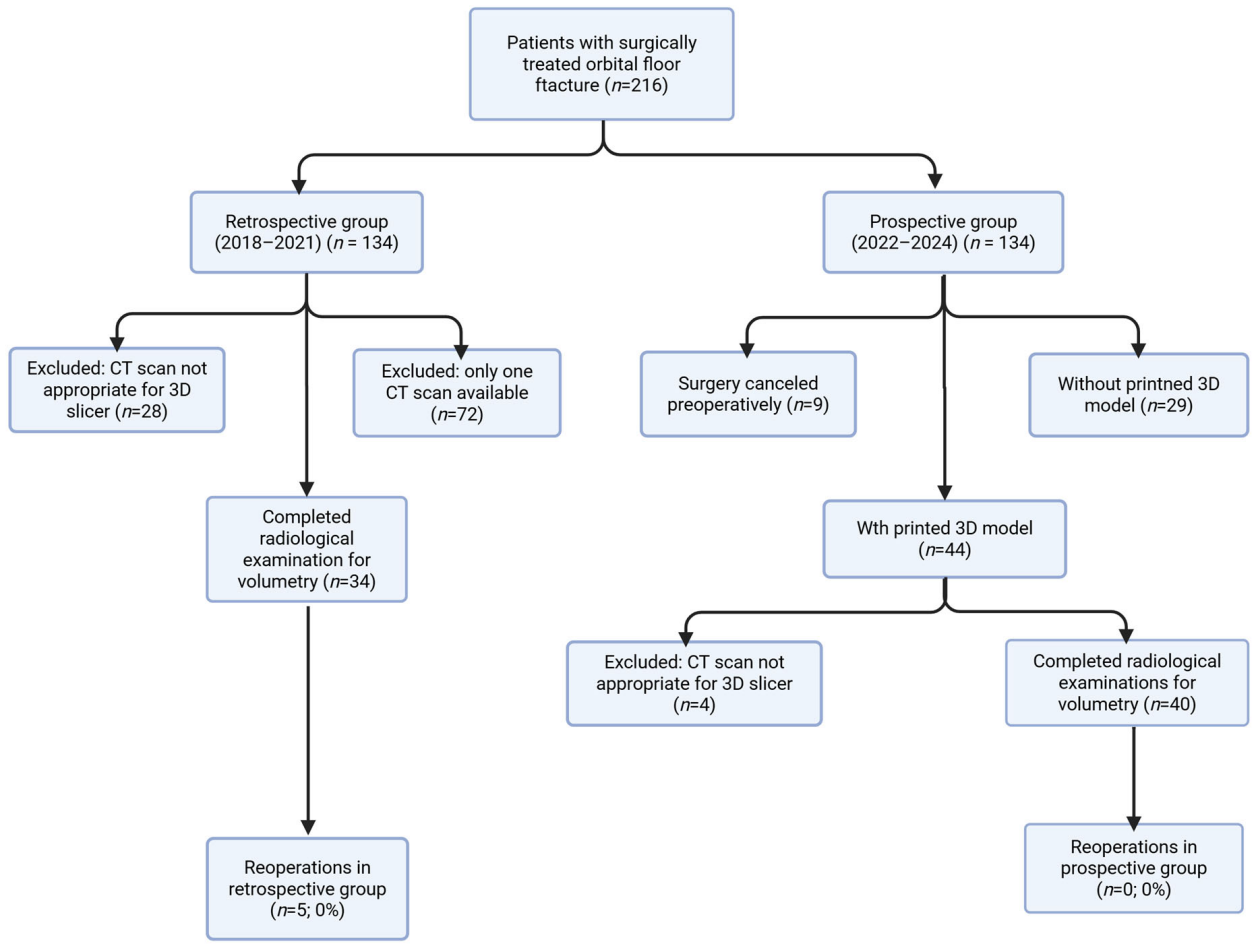
Patient flow chart.

Between the years 2018 and 2023, a total of 216 patients underwent surgical treatment for orbital floor fractures at Centre of oral and maxillofacial surgery, P. Stradins CUH, Riga, Latvia. Following the application of exclusion criteria (primarily due to inadequate CT scan quality or incomplete clinical records), 74 patients were included in the final analysis. The study population was divided into two cohorts: the retrospective (control) group (*n* = 34) and the prospective (study) group (*n* = 40) (Figure 5).

The mean age was 42.2 ± 13.5 years in the retrospective group and 45.4 ± 18.0 years in the prospective group. The majority of patients were male (79% in the retrospective and 57% in the prospective group). The left orbit was more frequently affected in both groups (56% and 55%, respectively). Regarding the timing of surgery, the median time to intervention was 10.5 days (IQR 5.5–24.8) in the retrospective group and 11.5 days (IQR 7.8–14.0) in the prospective group. The descriptive statistics are detailed in Table 1.

### 3.2. Clinical Outcomes and Reoperation Rates

Preoperative enophthalmos was observed in 10 patients (25%) in the prospective group and resolved following surgery. In the retrospective group, enophthalmos was not consistently documented in all patients. No eye motility disorders were identified either preoperatively or postoperatively, and no patients in the study cohort required emergency surgical intervention.

Preoperative diplopia was present in 20 patients (62%) in the retrospective group and 29 patients (73%) in the prospective group. Postoperative recovery patterns differed between the groups: retrospective group: diplopia persisted in 6 patients (18%) at 2 weeks, 5 patients (15%) at 2 months, and 4 patients (12%) reported persistent diplopia at the final 6-month follow-up. Prospective group: transient diplopia was observed in 16 patients (40%) at 2 weeks. This was reduced to 3 patients (8%) at 2 months. At the 6-month follow-up, persistent diplopia was noted in only 1 patient (3%). No statistically significant difference in diplopia rates was observed between the two groups at the 6-month follow-up (*p* = 0.114). Postoperative CT imaging and clinical assessment in the retrospective group revealed suboptimal mesh positioning in 5 patients (15%), leading to a secondary surgical intervention. In contrast, despite the single case of persistent diplopia, no patients (0%) in the prospective group required secondary surgical revision for implant repositioning. This reduction in the re-operation rate was statistically significant (*p* = 0.017).

### 3.3. Volumetric Analysis

**Figure 6:**
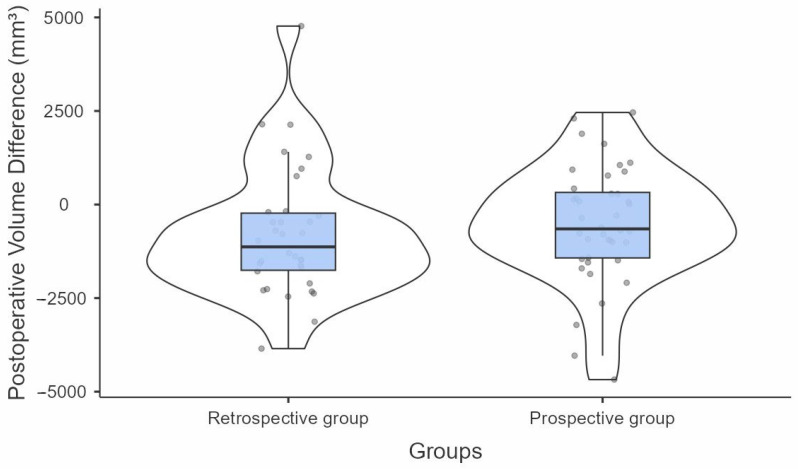
Comparison of postoperative orbital volume discrepancy (mm^3^) between the study groups.

The intra-rater reliability for orbital volume measurements was found to be good to very good (ICC: 0.771–0.965) [9]. The mean volume of the unaffected (healthy) orbit was similar in both groups (retrospective: 23,898 ± 2512 mm^3^; prospective: 22,756 ± 2814 mm^3^). In the retrospective group, the mean preoperative volume of the affected orbit was 26,186 ± 3739 mm^3^, which decreased to 23,107 ± 3156 mm^3^ postoperatively (a mean reduction of 3078 ± 2204 mm^3^). In the prospective group, the mean preoperative volume was 24,712 ± 3378 mm^3^, decreasing to 22,321 ± 2819 mm^3^ postoperatively (a mean reduction of 2390 ± 1893 mm^3^) (Figure 6).

**Figure 7:**
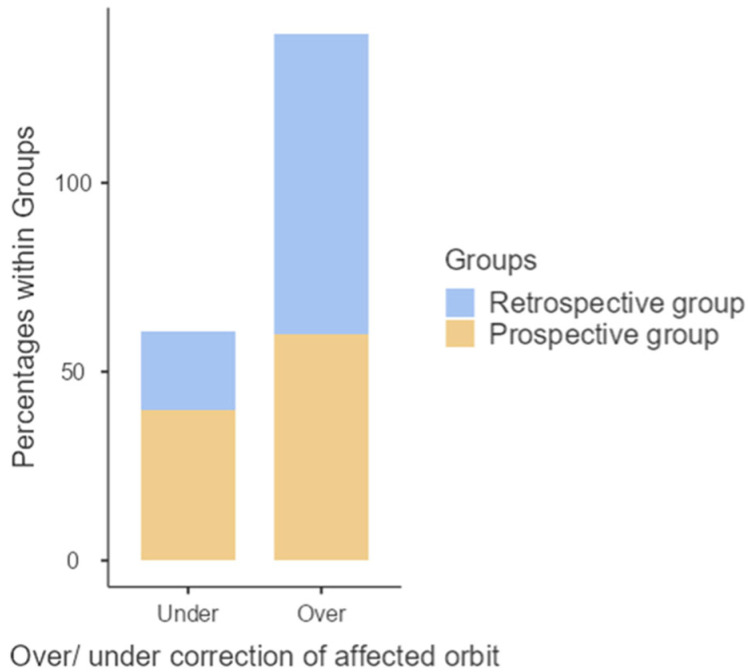
Distribution of postoperative orbital volume over- and under-correction between the study groups.

The accuracy of the reconstruction was assessed by comparing the postoperative orbital volume with the healthy contralateral side. Retrospective group: overcorrection was observed in 27 cases (79%) with a mean difference of 1479 mm^3^, while under correction occurred in 7 cases (20%) with a mean difference of 1921 mm^3^. Prospective group: overcorrection was noted in 23 cases (56%) with a mean difference of 1531 mm^3^, and under correction in 17 cases (44%) with a mean difference of 853 mm^3^ (Figure 7).

Although the prospective group showed smaller volume discrepancies (mean differences of 1479 mm^3^ vs. 1921 mm^3^ in overcorrected cases), no statistically significant correlation was found between postoperative orbital volume changes and the presence of diplopia in either group. Furthermore, no correlation was found between orbital volume and diplopia in the preoperative period.

## 4. Discussion

The management of orbital fractures represents a significant topic within the field of maxillofacial surgery. The treatment of orbital floor fractures remains challenging because of their complex anatomy and reduced visualisation during surgery [1,10]. If left untreated or treated improperly, they can lead to persistent enophthalmos and diplopia [11,12].

In this study it was observed that the use of in-house 3D-printed models for preoperative mesh forming significantly reduced the rate of need for revision surgeries. While the conventional intraoperative free-hand bending technique in our retrospective cohort resulted in a 15% reoperation rate due to implant malposition or persistent diplopia, the 3D-assisted protocol eliminated the need for secondary surgical intervention in the prospective group (*p* = 0.017). It provides a critical quality control step, ensuring that the implant fits the defect precisely before insertion. Our results align with recent literature advocating for technological aids in orbital surgery. A recent survey by Burger et al. (2024) among maxillofacial units in Switzerland, Germany, and Austria revealed that while 43% of surveyed clinics now have access to 3D printers in these countries, only a minority use them routinely, indicating a potential underutilization of this beneficial technology [6].

Consorti et al. (2024) demonstrated that pre-formed meshes can achieve accuracy comparable to patient-specific implants (PSIs) when used with navigation [13]. Although in our study no intraoperative navigation was used, we observed that 3D models allowed accurate mesh adaptation, offering a reliable alternative to expensive PSIs or intraoperative navigation systems, which may not be available in all institutions [3,14,15]. Similarly to Kallaverja et al. (2024) and a recent case–control study by Troise et al. (2026), who compared in-house 3D printing versus standard reconstruction, we found that the 3D workflow offers superior volumetric recovery and predictability [16,17]. While both of these author groups highlighted a reduction in surgical time with pre-bent meshes, our study specifically emphasizes the safety benefit—the elimination of the need for reoperation. This zero-reoperation rate is strongly corroborated by a recent case–control study by Troise et al. (2026), who reported that an in-house 3D preoperative workflow not only significantly reduced operative times but also eliminated implant malposition (0% vs. 25% in the conventional group) [17].

Abd El Ghafar et al. (2025) recently reported high long-term complication rates with titanium meshes, noting a reoperation rate of 31.8% due to issues like implant malposition and adherence syndrome when compared to porous polyethylene [2]. In contrast, our study demonstrates that titanium mesh can be used safely with a 0% reoperation rate, provided it is accurately pre-bent on a patient-specific 3D model. This distinction is crucial; it suggests that the material itself is not the sole cause of failure, but rather the intraoperative adaptation and positioning accuracy are the deciding factors. Furthermore, as shown in animal models by Guillaume et al. (2020), a titanium mesh typically undergoes fibrous encapsulation rather than direct osseointegration [18]. This lack of biological bonding makes precise anatomical friction-fit and screw fixation essential to prevent implant migration and soft tissue entrapment.

Regarding volumetric analysis, it was demonstrated that both techniques reduced the enlarged posttraumatic orbital volume to values close to the healthy opposite orbit. However, consistent with Fawzy et al. (2022) and Kallaverja et al. (2024), our study showed more favourable volumetric outcomes in the 3D group with less variance compared to the standard group [14,16]. We also observed a tendency toward slight overcorrection in both groups. As noted by McGurk et al. (1992), physiological asymmetry between healthy orbits can differ by up to 600 mm^3^ [12,18,19], suggesting that absolute mathematical symmetry is not always necessary for a successful clinical outcome.

Our study found no direct correlation between postoperative orbital volume and residual diplopia. In the retrospective group, 12% of patients had persistent diplopia at 6 months, whereas in the prospective 3D group, only one patient (3%) reported diplopia. In this specific 3D-assisted case, the persistent double vision was attributed to initial trauma resulting possibly in direct extraocular muscle injury and soft tissue healing and highlighting that functional recovery is not solely dependent on precise bony reconstruction. The lack of correlation suggests that postoperative double vision is multifactorial—dependent on soft tissue scarring, extraocular muscle entrapment, or nerve paresis—rather than bony volume alone [20,21]. This explains why transient diplopia was observed initially in the prospective group (likely due to dissection oedema) but resolved in the majority of cases without further surgery. From a practical standpoint, the in-house 3D printing workflow proved to be cost-effective and accessible. Unlike outsourcing PSIs, which can be costly and time consuming, in-house printing allows for rapid planning [10,22,23].

The study has several methodological limitations. First, the inclusion of a historical retrospective control group introduces an inherent risk of selection and attrition bias. In particular, long-term follow-up data within the retrospective cohort were incomplete, especially for patients without postoperative complications, thereby limiting the reliability of comparisons of subjective outcomes. Furthermore, the retrospective design precluded consistent and comprehensive documentation of detailed clinical histories, including secondary clinical parameters such as sensory deficits and standardized assessments of enophthalmos or exophthalmos. Considering these limitations, the study methodology was deliberately centred on objective, quantifiable radiological parameters to ensure a more robust and reliable comparison between the study groups. Additionally, the absence of clinical and intraoperative photographic documentation represents a further limitation, as it restricts the visual assessment of surgical outcomes. Nevertheless, despite its inherent constraints, the inclusion of a retrospective cohort remains of considerable scientific value. It enables the contextual comparison of historical diagnostic and therapeutic approaches with contemporary practices, thereby contributing to a more comprehensive interpretation of treatment evolution. Thus, even in the presence of incomplete data and methodological limitations, retrospective analyses can provide meaningful complementary insights that enhance the overall interpretability and external validity of the study findings. Second, we used a semi-automated segmentation method for volume calculation. Although manual segmentation is the gold standard, it is time consuming [4]. We found semi-automated segmentation to be reliable (high ICC) and efficient, as was described by Chepurnyi et al. (2020) and also Sentucq et al. (2021) [24,25], although defining the anterior border and fragmented inferior wall remained technically challenging. Finally, while the observed clinical benefits are highly significant, our sample size remains relatively small. To support these findings and establish guidelines for virtual surgical planning in orbital reconstruction, future large-scale, multicenter prospective randomized trials incorporating advanced 3D volumetric measurements are necessary.

## 5. Conclusions

The use of 3D-printed models for preoperative mesh adaptation significantly improves the safety and predictability of orbital floor reconstruction. This method reduced the re-operation rate to 0% in our cohort when compared to the conventional technique. While orbital volume restoration is achievable with standard methods, 3D planning offers a superior and cost-effective workflow that minimizes the risk of implant malposition and improves long-term clinical outcomes.

## Figures and Tables

**Table 1:** The baseline characteristics and demographic data of the patients.

	**Retrospective Group**	**Prospective Group**
Age (mean; SD)	42.2 (±13.5)	45.4 (±18.0)
Sex:		
Male	27 (79%)	23 (57%)
Female	7 (21%)	17 (43%)
Affected orbit		
Left	19 (56%)	22 (55%)
Right	15 (44%)	18 (45%)
Time to surgery, days, Median (IQR)	10.5 (5.5–24.8)	11.5 (7.8–14.0)
Preoperative enophthalmos	NA	10 (25%)
Preoperative diplopia	20 (62%)	29 (73%)
Postoperative diplopia		
1–2 weeks after surgery	6 (18%)	16 (40%)
2 months after surgery	5 (15%)	3 (8%)
6 months after surgery	4 (12%)	1 (3%)
Number of reoperations	5 (15%)	0 (0%)

NA—not applicable, not clearly documented in retrospective group.

## Data Availability

The data presented in this study are available on request from the corresponding author. (The data presented in this study are not publicly available due to privacy and ethical restrictions).

## Abbreviations

The following abbreviations are used in this manuscript:

3D	Three-dimensional
PSI	Patient-specific implants
VSP	Virtual surgical planning
CT	Computer tomography
CUH	Clinical University Hospital
DICOM	Digital Imaging and Communications in Medicine
ICC	Intraclass correlation coefficient
